# Nephrin autoimmunity: signal, noise, and a path to clarity

**DOI:** 10.1172/JCI208243

**Published:** 2026-07-01

**Authors:** Dhruti P. Chen, Ronald J. Falk

**Affiliations:** 1 Department of Medicine, UNC School of Medicine, University of North Carolina, Chapel Hill, North Carolina, USA.; 2 UNC Kidney Center, Chapel Hill, North Carolina, USA.

## Abstract

Advances in antigen discovery and autoantibody profiling have reshaped the classification of autoimmune kidney diseases, moving beyond purely histologic definitions. The identification of podocyte-targeting autoantibodies has transformed the understanding of nephrotic syndrome, prompting renewed interest in autoimmune mechanisms underlying podocytopathies. Recent reports of nephrin autoantibodies in minimal change disease, the most common cause of nephrotic syndrome in children, suggested a potential antigen-defined subset, but findings have been inconsistent. In this issue of the *JCI*, Pecoraro and colleagues advance the field by systematically interrogating anti-nephrin antibodies across a diverse nephrotic syndrome cohort using human-based and orthogonal approaches. Their results highlight critical limitations in assay specificity and cohort heterogeneity while raising the question of the clinical utility of anti-nephrin antibodies in the care of patients with minimal change disease. More broadly, this study underscores the need for collaboration to establish standardized assays and rigorously phenotyped cohorts.

## From idiopathic to autoantibody-defined kidney disease

Gone are the days when many kidney diseases were described as idiopathic or “primary.” Many of these so-called idiopathic diseases are now recognized as having an underlying autoimmune etiology. This paradigm shift — from histologic descriptors to antigen and autoantibody-based disease definitions — has clarified clinical decision-making and enabled targeted therapy. For example, what was called Goodpasture syndrome is now understood to be an autoantibody-mediated disease directed largely against a single antigen, in which antibodies to the α3 chain of type IV collagen cause fulminant crescentic glomerulonephritis (1). In anti-neutrophil cytoplasmic autoantibody–associated (ANCA-associated) glomerulonephritis and ANCA-associated vasculitis, antibodies to myeloperoxidase (MPO) or proteinase-3 (PR3) activate neutrophils and monocytes, leading to pauci-immune necrotizing glomerulonephritis (2–4). Accordingly, classification of either disease can now be based on MPO- and PR3-ANCA serotypes.

Membranous nephropathy has become the prototype of organ-specific kidney autoimmunity with the discovery of podocyte antigens such as PLA2R and THSD7A and correlation of antibody levels against these antigens with disease activity (5, 6). More recently, mass spectrometry–based approaches have expanded the antigen spectrum to almost 30 different antigens (e.g., EXT1/EXT2, NELL-1), blurring the distinction between what was previously designated as “primary” membranous nephropathy and autoimmune-associated forms (7, 8). The discovery that galactose-deficient IgA1 molecules with resultant autoantibodies drive IgA glomerulonephritis pathogenesis has fueled the development of therapies targeting B cell activating factor and transmembrane activator and CAML interactor (TACI) signaling in B cells (9–13). Overall, these advances establish autoantibodies as central drivers, biomarkers, and therapeutic targets across autoimmune kidney diseases.

## Linking nephrin autoantibodies to immune-mediated podocytopathies

In children, nephrotic syndrome most commonly results from minimal change disease, which shows essentially normal glomeruli on light microscopy. In many very young children, a kidney biopsy is deferred because patients diagnosed with so-called “idiopathic nephrotic syndrome” often show a brisk response to glucocorticoid therapy. Some subsequently experience relapsing disease, whereas others become steroid dependent or resistant. A subset of these children (and some adults) who demonstrate a brisk response to glucocorticoids also exhibit a remarkable and sustained response to B cell–depleting therapies (14). It is therefore unsurprising that autoantibodies have been implicated in disease pathogenesis. The elusive question has been to identify the antigen or antigens driving this autoimmune response.

With considerable excitement, several studies identified circulating autoantibodies against nephrin in a subset of patients with minimal change disease. Nephrin is a critical structural component of the slit diaphragm of the glomerulus, where it maintains filtration barrier integrity (15). Mutations in the nephrin gene (*NPHS1*) cause congenital nephrotic syndrome (16). In minimal change disease, autoantibodies to nephrin appear to track with disease activity and correspond to punctate podocyte IgG colocalization with nephrin on kidney biopsy, suggesting direct in situ antibody binding (17). Subsequent multicenter work showed that anti-nephrin antibodies correlate with disease activity and are potentially pathogenic (18, 19). Importantly, when applied to pediatric cohorts stratified by treatment response, anti-nephrin antibodies were largely confined to steroid-responsive disease and nearly absent in genetic or multidrug-resistant nephrotic syndrome. This observation refines the clinical relevance of anti-nephrin antibodies and argues against a universal role for them across podocytopathies (20). Collectively, these findings support the concept that nephrin autoantibodies contribute to disease pathogenesis in a defined subset of immune-mediated podocytopathies.

## Methodological and cohort heterogeneity challenges nephrin autoimmunity as a unifying paradigm

The concept of nephrin autoimmunity in podocytopathies has evolved rapidly but remains controversial. This controversy is driven largely by limitations of available antibody assays and differences in patient cohort composition. In this issue of *JCI*, Pecoraro and colleagues looked for nephrin autoantibodies in a heterogeneous cohort spanning steroid-sensitive and -resistant disease, adult and pediatric populations, and multiple post- or pretransplant states (21). Their findings suggest that anti-nephrin autoantibodies do not represent a unifying pathogenic mechanism or a reliable biomarker across podocytopathies; indeed, anti-nephrin autoantibody positivity was highest in the control group of healthy volunteers when using murine-derived nephrin. The authors found that high seropositivity to murine-derived nephrin largely resulted from cross-reactivity with natural anti–α-galactose antibodies, rather than reflecting true anti-nephrin autoimmunity. Using human-derived nephrin, immunoprecipitation, and high-resolution kidney biopsy imaging, they demonstrated a paucity of circulating or tissue anti-nephrin IgG.

These data challenge findings from prior studies that used heterogeneous cohorts and differing methodologies (17, 18, 22–25). These studies tested the antibodies with ELISA studies and Western blots, incorporating immunoprecipitation products in slightly varied methods. The extracellular domain of nephrin was utilized as the antigen, either from mouse (23, 24) or human cells (17, 18). Liu et al. first identified discrepancies between murine- and human-derived nephrin antigens (25). Building on this, Pecoraro et al. examined the issue more systematically, demonstrating that human-derived nephrin is a reliable source for testing (21). Their study is notable for its inclusion of both pediatric and adult patients while excluding genetic etiologies, whereas prior reports have applied variable genetic stratification approaches in predominantly pediatric or mixed cohorts (17, 18, 20, 22). These are just a few of the differences between studies to underscore the lack of a standardized, universally applicable method for detecting anti-nephrin autoantibodies across nephrotic syndrome populations.

## Resolving the controversy through standardization and collaboration

How, then, should this controversy be resolved? The challenges of assay development should be recognized as an expected — and surmountable — feature of early biomarker science. It took many years to standardize what are now routine MPO-ANCA and PR3-ANCA serological tests. Large-scale screening using standardized, consensus methodologies are essential to distinguish true biological signal from technical artifacts. Similarly, patient cohorts must be more rigorously defined and standardized to assess the validity of anti-nephrin autoantibodies as a biomarker. Clinically, in nephrotic syndrome, we observe heterogeneity in age, glucocorticoid responsiveness, disease duration, and other variables that remain poorly characterized. It is likely that patients with minimal change disease who respond to B cell–depleting therapy harbor multiple potential pathogenic antigens. Analogous to membranous nephropathy, successive discoveries will likely reveal varied antigenic targets rather than a single unifying antigen. Continued, unbiased exploration of additional antigens and autoantibodies therefore remains essential. Once a rigorously validated assay becomes widely available, its broad application to nephrotic populations will enable real-world, experience-driven refinement of disease definitions, antibody kinetics, and clinical relevance.

Ultimately, questions of antigen selection, patient stratification, and assay validity are best resolved through careful experimental work at the bench, rather than debate at the podium. Resolving the current controversy will require direct collaboration rather than parallel inference, including reagent sharing, assay cross-validation, and transparent head-to-head testing across laboratories using shared cohorts. To better serve our patients, the glomerular disease community must remain committed to uncovering the full spectrum of autoimmune responses driving kidney disease. Only then can we consign terms such as “idiopathic” or “primary” — which are unsettling and uninformative for patients and clinicians alike — to the rearview mirror.

## Conflict of interest

The authors have declared that no conflict of interest exists.

## Funding support

This work is the result of NIH funding, in whole or in part, and is subject to the NIH Public Access Policy. Through acceptance of this federal funding, the NIH has been given a right to make the work publicly available in PubMed Central.

- National Institute of Allergy and Infectious Diseases (K08AI187710 to DPC).
- National Institute of Diabetes and Digestive and Kidney Diseases (U01DK100867 to RJF).

## Version 1 07/01/2026

Electronic publication
